# The impact of age on right ventricular morphology and function late after repair of Tetralogy of Fallot: a cardiac magnetic resonance study

**DOI:** 10.1186/1532-429X-16-S1-P125

**Published:** 2014-01-16

**Authors:** Benedetta Leonardi, Marcello Chinali, Aurelio Secinaro, Emanuela Valsangiacomo Buechel, Valentina Silvestri, Sonia B Albanese, Adriano Carotti, Giacomo Pongiglione

**Affiliations:** 1 Department of Cardiology and Cardiac Surgery, Bambino Gesu' Children's Hospital IRRCCS, Rome, Rome, Italy; 2 Department of Diagnostic Imaging, University Children's Hospital, Zurich, Switzerland

## Background

Cardiac magnetic resonance (CMR) is the gold standard to evaluate right ventricular (RV) hemodynamics after repair of Tetralogy of Fallot (TOF). CMR markers, such as severe RV dilation and biventricular dysfunction, are independent predictors of death, sustained VT and heart failure. However, the relationship between patient's age, type and time of corrective surgery, severity of PR and RV/LV dimensions and function has not been fully explained.

## Methods

We enrolled patients who underwent hemodynamic evaluation by CMR late after TOF repair (by either transannular or infundibular RV outflow tract reconstruction) in two pediatric cardiology centers, between March 2008 and March 2013. Surgical data (time and types of repair) and CMR parameters were collected.

## Results

The study included 208 patients (62% males) aged 17.5 ± 6.6 (range 5-38) years, with age at repair 16.3 ± 21.7 months (range 0.2-146 months). RV end-diastolic volume indexed (RVEDVi) was mildly negatively correlated with patient's age (figure; p = 0.019, r = -0.163). In details the negative correlation between RV dilation was stronger when limiting the analysis to patients older than 20 years (figure). On the contrary, LVEDVi was mildly positively related to patient's age (p = 0.020, r = 0.161). RVEDVi was also correlated with time interval from corrective surgery, PRF and RVOT obstruction (all p < 0.05). Eventually, older age at repair and RVEDVi significantly affected RVEF (all p < 0.05). LVEF was significantly correlated with RVEF (p < 0.001) and the time elapsed from the repair (p = 0.016, r = 0.171).

## Conclusions

RV dilation in repaired TOF patients might have an unexpected behavior over time, with older patients showing a progressive reduction in RV volume. Longitudinal follow-up studies are needed to verify our findings and define the role of a hypothetical RV "restrictive physiology" in predicting the adverse outcomes of patients with repaired TOF.

## Funding

No disclosure to make.

